# Chiropractic case reports: a review and bibliometric analysis

**DOI:** 10.1186/s12998-021-00374-5

**Published:** 2021-04-28

**Authors:** Robert J. Trager, Jeffery A. Dusek

**Affiliations:** Connor Integrative Health Network, Cleveland Medical Center, 11000 Euclid Ave, Cleveland, OH 44106 USA

**Keywords:** Case reports, Bibliometrics, Literature review, Chiropractic, Spine, Musculoskeletal diseases, Information science

## Abstract

**Objective:**

To determine publication trends, gaps, and predictors of citation of chiropractic case reports (CRs).

**Methods:**

A bibliometric review was conducted by searching PubMed, Index to Chiropractic Literature (ICL), and Google Scholar to identify PubMed-indexed CRs, which were screened according to selection criteria. Case reports were categorized by International Classification of Disease (ICD-10) code, patient age, topic describing case management or adverse effects of care, focus being spinal or non-spinal, journal type, integrative authorship, title metrics, and citation metrics. Binary logistic regression was used to identify independent predictors of citations per year and total citations greater than the median values.

**Results:**

The search identified 1176 chiropractic CRs meeting selection criteria. There was an increasing trend of CRs having a case management topic, non-spinal focus, non-chiropractic journal, neuromusculoskeletal-focus, diagnosis of vascular pathology, and a decreasing trend of adverse effect vascular pathology CRs. Independent predictors of greater total citations (or citation rate) included ICD-10 categories of perinatal conditions, infections, “case” in title, case management topic, and physical therapy, integrative, and dental journal type. Predictors of fewer citations included diseases of the blood, neoplasms, other findings not elsewhere classified, a title > 11 words, and multidisciplinary authorship. ICD-10 categories describing non-musculoskeletal diseases and special populations such as pediatrics, pregnancy, and perinatal conditions had few CRs.

**Conclusion:**

Chiropractic CRs are diversifying from spine-related topics. Chiropractors are encouraged to publish objective, structured CRs within defined research gaps. Published CRs can inform the design of future research studies with a higher level of clinical relevance and evidence.

**Supplementary Information:**

The online version contains supplementary material available at 10.1186/s12998-021-00374-5.

## Introduction

Bibliometrics is a quantitative study of research publications, often using statistical methods. Case reports (CRs), also called clinical CRs, are uncontrolled description of the clinical course of one to five patients [1, 2]. Some authors distinguish a CR from a case study, the latter of which has detailed measurements over time [3]. There are few bibliometric reviews of chiropractic research and these have typically focused on publications within a single journal [4–8]. The most recent and comprehensive bibliometric study of peer-reviewed chiropractic CRs was published in 1993 and identified 318 CRs [9].

Chiropractic CRs typically focus on either diagnosis or treatment of a patient or have an educational focus [10]. Diagnosis-focused CRs illustrate how a chiropractor evaluated or assessed a rare or confusing presentation [10], while treatment-focused CRs document improvement in a patient’s condition as objectively as possible [11]. This review includes a fourth category of CRs which describe adverse effects of chiropractic care, the inclusion of which will aid in the completeness of the bibliometric analysis.

### Chiropractic interest in CRs

Although few chiropractors conduct research, many are interested in doing so. One United States-based study found that 27% of chiropractic students planned on conducting and publishing some form of research [12], and a 2011 survey of the American Chiropractic Association members found that 60% reported an interest in conducting research [13]. Despite this interest, only about 2–6% of chiropractors internationally have published or are engaged in research activities [14–16]. Notwithstanding, two reviews specific to chiropractic journals found that CRs were the most frequently published form of chiropractic research [5, 6] and thus may serve as an entry point into research for chiropractors.

Accordingly, multiple chiropractic editorials and commentaries have encouraged students and clinicians to publish CRs [8, 11, 17–25]. These and other publications have provided detailed instructions for chiropractors on how to write CRs [8, 10, 11, 26–29]. Authors promoting chiropractic CRs have described motivations to publish CRs that are more specific to the profession which include:

- To increase the chiropractic research database [10, 19, 27]
- To increase awareness of chiropractic in the scientific and healthcare community [19]
- To improve inter-professional relationships [19]
- To stimulate intra-professional collaborations [22]
- Important clinical observations are going unpublished [22] or treatment effects only have anecdotal support [9, 11, 30]
- To present the case management of non-musculoskeletal, non-spinal disorders [17, 18]
- To focus on populations with a limited research base such as pediatric patients [18, 25] and pregnant women [25]
- To help other clinicians with similar cases [11, 18, 21, 22, 25]

### Strengths of this review

To our knowledge, no prior studies have subcategorized a broad range of chiropractic CRs or investigated predictors of chiropractic CR citation rates.

### Purpose of this review

The aims of this review are to:

1. Identify trends and gaps in the research for chiropractic CRs by examining ICD-10 disease category, patient age, topic being case management or adverse effects, focus being spinal or non-spinal, journal type, and integrative authorship.
2. Summarize citation data for chiropractic CRs with reference to the above listed variables and title metrics.
3. Perform a multiple logistic regression to identify independent variables associated with greater citations of chiropractic CRs.

## Methods

### Search strategy

Test searches were conducted using PubMed, Microsoft Academic Search, the Index to Chiropractic Literature, EbscoHost, Google Scholar, and Google Scholar using Harzing’s Publish or Perish 7 (Tarma Software Research Ltd.®) to refine the search terms and identify the most accurate and time-saving search strategy. Prior researchers have recommended searching both PubMed/MEDLINE® and a chiropractic database (ICL or MANTIS/Chirolars) when conducting chiropractic research because no single database will return all relevant results [31].

In test searches, PubMed returned fewer CRs than expected based on the amount indexed in ICL. Certain CRs that appeared in ICL, but not the PubMed test searches, could be secondarily identified on PubMed by directly searching for the CR title. Conversely, there were CRs in PubMed that did not appear in ICL, such as those published in general medical journals, CR journals, or physical therapy journals.

A preliminary search of EBSCOhost was performed to compare the number of CRs relative to other health disciplines and these CRs were not screened for inclusion into the review. This database enables sorting CRs by specialty. Using the “Advanced Search” options, under “Publication Type” the option “Case Study” was selected (which includes CRs), and results were searched for each category under the “Special Interest” section on 8/25/2020.

Ultimately, three databases were searched for inclusion of CRs into this review: PubMed, ICL, and Google Scholar (Table 1). Search results were downloaded to comma-separated value files and combined into a Microsoft Excel workbook. Articles excluded with specific reasons were recorded in a separate sheet.

**Table 1:** Search strategy

Database	Date of search	Criteria	Initial results
Index to chiropractic literature (ICL)	10/30/2020	Article type: Case report, Peer Reviewed	2204
PubMed	10/30/2020	((chiropract*[Title/Abstract]) OR (chiropract*[Affiliation])) OR (chiropractic [MeSH Terms]) Article type: Case report	772
Google Scholar using Harzing’s Publish or Perish	10/30/2020	Keywords: “case report” chiropractic	First 100 results screened
EBSCOhost	8/25/2020	Publication type: Case study Special interest: Chiropractic Care	609

The search of ICL was performed to identify PubMed-indexed CRs that were not found in the initial PubMed search. The advanced search function was set to Article type: “Case report,” and “Peer Reviewed articles.” Articles were included provided they were indexed on PubMed and met the other selection criteria.

### Selection criteria

A broad range of types of CRs were included in this study, as described in a prior article [3], including retrospective cases, N-of-1 studies, single subject trials, and case studies, provided they met the selection criteria listed below. The broad range of study descriptions in our inclusion criteria was purposeful and intended to account for variation in terminology used to describe such cases. In addition, the terms “case study” and “case history” map to “case reports” in Medical Subject Headings in PubMed [3] and were identified using the search strategy. Additional inclusion criteria were the reporting of (1) patient age, (2) sex and (3) relation to any aspect of chiropractic diagnosis, treatment, or case management.

Exclusion criteria were studies that had more than 5 patients, non-human subjects, were anatomical-cadaver studies, and mentioned chiropractic but were unrelated to chiropractic diagnosis or management, or the association was unclear. This criterion was necessary as previous research has highlighted that the terms “chiropractor” or “chiropractic manipulation” are often misused in published CRs describing adverse responses to therapies [32, 33]. This review utilized these prior publications to aid in the exclusion of CRs that used inappropriate terminology. Studies that examined the mechanisms of a diagnostic or treatment method on asymptomatic volunteer(s) were also excluded. Utilization of a quality checklist such as the CARE (CAse REports) guidelines [34] was beyond the scope of this review.

PubMed indexing of CRs was a prerequisite in this study as a means of standardizing the selection of CRs. Although there is an overlap of “peer-reviewed” and “PubMed-indexed” CRs, some chiropractic journals publish peer-reviewed CRs that are not PubMed indexed, for example the *Journal of Clinical Chiropractic Pediatrics*.

### ICD-10 category

Grouping CR disease by ICD-10 blocks [35] (Table 2), also called categories, has been recommended as a means to categorize CRs for further analysis [36]. Case reports were assigned the best-matching ICD-10 category code [37] which was identified by entering the primary CR diagnosis into a search engine [38]. When there was no exact match for a diagnosis, a Google search was performed to find the most applicable ICD-10 code. When a patient presented with spinal complaint and another systemic complaint, the non-spinal complaint took precedence and was cataloged by ICD-10 category. For CRs describing an adverse effect of chiropractic care, the resulting effect was coded (e.g. fracture) rather than the patient’s presenting complaint (e.g. headache) or V code (external causes of morbidity and mortality). For case series with up to 5 patients having heterogeneous ICD-10 diagnoses, the most frequent ICD-10 category was used. Because there is no specific ICD-10 code for cervicogenic vertigo, CRs describing improvement of dizziness or vertigo after spinal manipulation were grouped within H60–H95. Cases describing “sports hernia” relating to tendinopathy were coded in the M00-M99 category, and those relating to a muscle tear in the S00-T98 category.

**Table 2:** ICD-10 categories used in this bibliometric analysis

Block	Title
A00–B99	Certain infectious and parasitic diseases
C00–D48	Neoplasms
D50–D89	Diseases of the blood and blood-forming organs and certain disorders involving the immune mechanism
E00–E90	Endocrine, nutritional and metabolic diseases
F00–F99	Mental and behavioural disorders
G00–G99	Diseases of the nervous system
H00–H59	Diseases of the eye and adnexa
H60–H95	Diseases of the ear and mastoid process
I00–I99	Diseases of the circulatory system
J00–J99	Diseases of the respiratory system
K00–K93	Diseases of the digestive system
L00–L99	Diseases of the skin and subcutaneous tissue
M00–M99	Diseases of the musculoskeletal system and connective tissue
N00–N99	Diseases of the genitourinary system
O00–O99	Pregnancy, childbirth and the puerperium
P00–P96	Certain conditions originating in the perinatal period
Q00–Q99	Congenital malformations, deformations and chromosomal abnormalities
R00–R99	Symptoms, signs and abnormal clinical and laboratory findings, not elsewhere classified
S00–T98	Injury, poisoning and certain other consequences of external causes
V01–Y98	External causes of morbidity and mortality
Z00–Z99	Factors influencing health status and contact with health services
U00–U99	Codes for special purposes

Publications for each ICD-10 block were grouped by year. These values were then graphed by percentage of publications as well as publication volume. The slope of the resulting trend line was used to identify publication trends, as has been done in prior studies [39, 40].

### Pediatric cases

Case reports were defined as pediatric if the described patient(s) were age 16 or younger, to be consistent with the U.S. Food and Drug Administration definition of a pediatric range for research purposes [41]. For CRs with multiple patients, the most common age bracket of pediatric vs. non-pediatric was used. If there were an even number of cases (1 or 2 each) of pediatric and non-pediatric, the pediatric determination was given.

### Adverse effects vs. case management topic

Case reports were divided into two topics, (1) case management-type, which included a diagnostic workup, clinical reasoning, or treatment approach, including integrative CRs, and (2) adverse effects, which included missed or delayed diagnoses resulting in an adverse event, or more direct adverse events related to chiropractic care such as treatment-related injury. These data were then graphed by publication year.

### Spinal versus non-spinal focus

Case reports were divided into two groups, (1) those focusing on spine-related or sacrococcygeal-related symptoms and/or treatments, and (2) those that focused on extraspinal conditions, outside of the spine and sacrococcygeal region. Cases of infection, tumors, or other diseases presenting with spine or sacrococcygeal symptoms were considered spine-related. Cases of vertebral artery pathology or abdominal aortic aneurysm causing neck or back pain were considered spine-related. Cases of shoulder and hip pathology in which treatment included spinal manipulation were also considered spinal. Cases pertaining to rib symptoms or costochondritis that did not involve therapies directed to the costovertebral or costotransverse joints were considered extraspinal.

### Citation metrics

Case report titles were entered into Google Scholar to calculate the total number of citations. This was done manually from 11/2/2020–11/3/2020. Citations per year were calculated using the formula [citations/(2020-(publication year))] as has been done in other studies [42]. Citation metrics were used from Google Scholar as opposed to another database (such as PubMed) because a larger scope of citation data was preferred to thoroughly examine the overall citation impact of CRs. Google Scholar records citations from a broader range of sources including the grey literature as well as book citations, and finds nearly all citations identified by Web of Science and Scopus [43].

### Journal metrics

Journal names were extracted from the database of selected CRs, sorted alphabetically, and duplicates were removed. Journals were cataloged using a code based on the focus of the journal, which was chosen based on the journal website description and/or mission statement. This system grouped journals into the following categories: medical, chiropractic or alternative and complementary medicine, radiology, physical therapy, integrative, CR, dental, and other. Neurology, surgery, and family medicine journals were cataloged as medical journals. Neuroradiolgy and diagnostic ultrasound journals were grouped with radiology journals. Journal H-index values were identified using SCImago Journal & Country Rank [44]. This value correlates with the Impact Factor (IF) and SCImago Journal Rank indicator (SJR) [45] and was used because certain journals did not have an assigned SJR or IF, while the H-index is freely available and can be easily found or calculated [46].

Author names were extracted from the database of selected CRs by delimiting to surname and initial(s), and duplicates were deleted. Some author names had multiple variations which was manually corrected (e.g. Chu ECP and Chu EC). Precautions were taken to avoid combining distinct authors (e.g. Jocelyn Cox and James M. Cox).

Further data were gathered for authors publishing in the top ten list of publication volume. Author H-indices were identified on 11/2/2020 using the authors’ Google Scholar profile. When this was not available, the author name was searched using Google Scholar via Harzing’s Publish or Perish tool with the additional keyword “chiropractic,” as these authors had a chiropractic degree or affiliation. Affiliations were extracted from the CR abstract or full-text.

### Integration metrics

Author degrees were identified for all non-chiropractic authors by searching the full-text of the CR, cross-referencing the author name with other publications, ResearchGate, ICL, or the researcher’s website or academic institution. Authors with a medical degree, but no identifiable specialty were recorded as having an “unlisted specialty.” In instances where an author obtained a non-chiropractic health degree after the CR publication date, the degree was not listed.

Case reports were recorded as having integrated authorship or single-discipline authorship. To be considered “integrated,” authors had to have different health care-related degrees related to patient care. These included medical and osteopathic degrees as well as allied health professions (e.g. physical therapy, nursing). A PhD author was not considered integrated for the purposes of this review. Case reports by a single author with dual degrees (e.g. DC and MD) were not considered integrated. Multi-author papers with at least one DC and a DC author who had an additional degree such as an RN or MD were considered integrated.

### Title metrics

The number of words and presence of a colon punctuation in each CR title was recorded using Excel counting formulas. Title length and colon punctuation has been identified by prior research to impact citation metrics [47]. Case reports were denoted that used the word stem “case” in the title or any of its derivations including “cases,” “case study,” or “case report,” a measure based on the CARE guidelines [34].

### Keywords

A key phrase finder [48] was used to identify occurrences of common 2-word phrases, to further clarify CR topic and focus.

### Logistic regression

Bivariate logistic regression was performed using GNU PSPP Statistical Analysis Software version 1.0.1. Regression is recommended as the most powerful method of identifying variables affecting article citations [49]. Logistic regression can be used when the distribution of citations is non-normal, to predict papers belonging to a highly cited group [49]. A cutoff *p*-value of 0.05 was used to denote statistical significance.

Prior to logistic regression, testing was performed for bivariate correlation using a two-tailed Pearson correlation matrix to identify strongly associated variables. None of the variables had a correlation coefficient of 0.7 or higher, except citations and citations per year (0.7, *p* <  0.001), which were analyzed in separate regression models. In addition, the number of (1) total citations and (2) citations per year were tested for a normal distribution by creating a histogram and by using the Kolmogorov–Smirnov test, which yielded an Asymp. sig (2-tailed) of < 0.001 for both, indicating a non-normal distribution.

Prior research on citation impact has recommended comparing the number of citations to the median value [50]. Other research has suggested using citations per year rather than total citations, to adjust for differences in publication year [42]. We used two models with a different independent variable for each: (1) > 7 total citations (> median value), and (2) >  0.6 citations per year (> median value). Odds ratios (ORs) with confidence intervals were calculated for each variable.

The following covariates were included in the logistic regression: ICD-10 category, pediatric subject, adverse effect or co-management topic, spinal or non-spinal focus, journal type, integrative authorship, and title metrics including presence of a colon (:), presence of the stem “case,” and number of words > 11.

## Results

### Search results

**Fig. 1:**
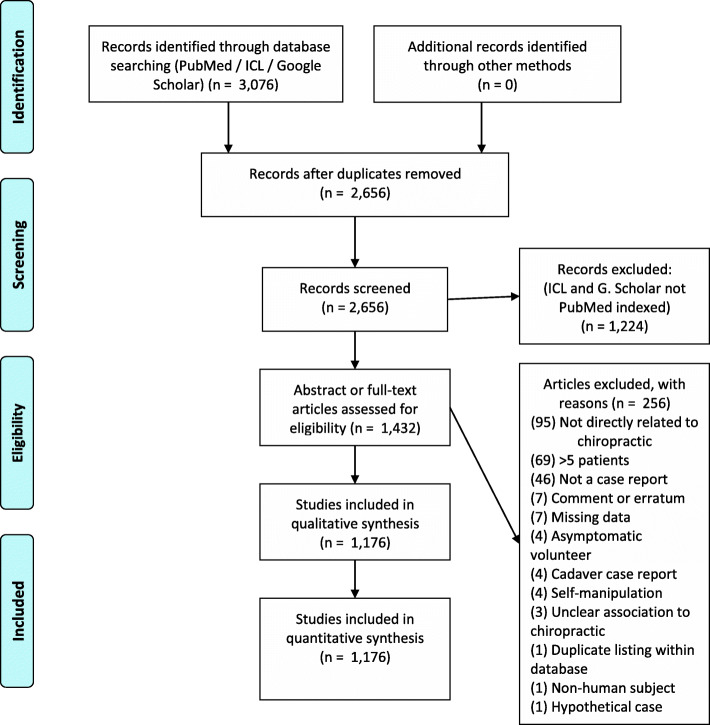
Preferred Reporting Items for Systematic Reviews and Meta-Analyses (PRISMA) flowchart

The search strategy identified 3076 records which was reduced to 1176 after the removal of duplicates and application of selection criteria (Fig. 1). The final list of CRs and those excluded by screening are available upon request.

**Fig. 2:**
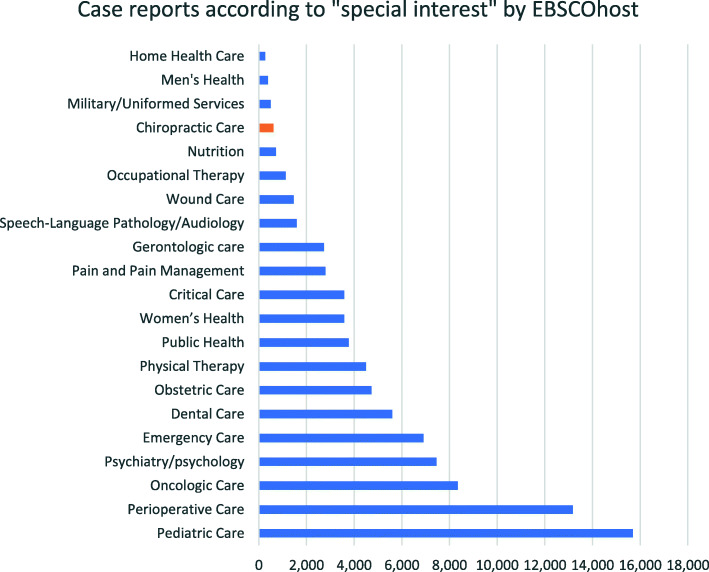
Case report volume indexed by EBSCOhost according to special interest on August 25th, 2020. Select interest groups are shown. Nursing language / classification, case management, nursing administration, nursing education, informatics, and quality assurance are not shown, which had fewer CRs than chiropractic, while evidence-based practice, advanced nursing practice, palliative/hospice care, and social work are also not shown, which had a greater number of CRs than chiropractic

There were fewer chiropractic CRs relative to other health disciplines or special interests as indexed by EBSCOhost (Fig. 2). For example, chiropractic CRs were outnumbered by pediatric care CRs by a factor of 25:1. This comparison had limitations in that EBSCOhost does not provide a comprehensive list of healthcare specialties, some chiropractic-authored CRs could have been categorized under physical therapy or other special interests, and this database returned fewer chiropractic CRs than PubMed or ICL.

### ICD-10 category

**Fig. 3:**
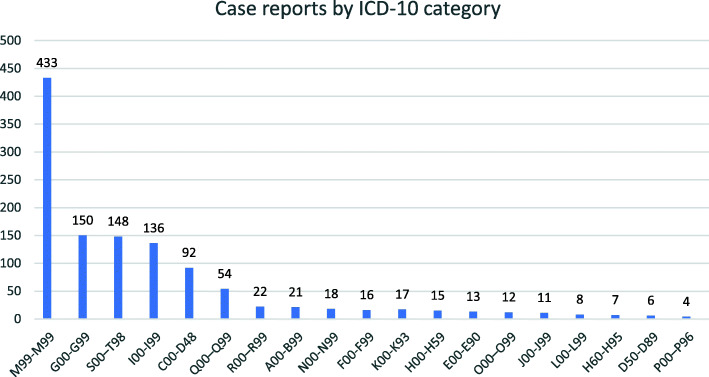
Volume of chiropractic case reports by ICD-10 category

Diseases of the musculoskeletal system and connective tissue (M00-M99) had the greatest volume of CRs (Fig. 3), representing 37.7% of identified CRs. Consistent with this finding was that the most common 2-word phrases had musculoskeletal associations including “back pain” (87 occurrences), “low back” (70), and “cervical spine” (46). The next most common phrase was “vertebral artery” (37 occurrences) which belonged to Diseases of the circulatory system (I00-I99). There were no CRs in the ICD-10 blocks V01–Y98, Z00–Z99, and U00–U99.

Certain conditions originating in the perinatal period (P00-P96) were represented by four CRs, the least of any category, with the first published in 1993, describing treatment for Erb’s palsy [51]. Others in this category described chiropractic management of breast-feeding difficulties [52] and gastroesophageal reflux in an infant [53]. Conditions of pregnancy, childbirth and the puerperium (O00-O99), a related ICD-10 category, likewise had few CRs. The first, published in 1995, was written by nurse midwife and described successful chiropractic manipulation of the pelvis to treat sciatica which developed during labor [54]. Other CRs in this category described the management of pregnancy-related pubic symphysis pain [55], lumbar radiculopathy [56], and meralgia paresthetica [57].

### ICD-10 trends

The rank of publication trends for the top two for case management and adverse effects CRs remained identical when trends for publication volume were analyzed rather than publication percentage, which is what is presented.

The ICD-10 categories with the greatest positive publication trend for case management CRs were Musculoskeletal and connective tissue diseases (M00-M99), followed by Injury, poisoning and certain other consequences of external causes (S00-T98) and Diseases of the nervous system (G00-G99) (Table 3). These categories contain neuromusculoskeletal diagnoses, including spinal pain and radiculopathy (M codes), myelopathy (G codes) and sprains and strains (S00-T98).

**Table 3:** Trend in ICD-10 category grouped by CR topic, as a percentage of total articles in each respective topic

Case management			Adverse effect	
Block	Slope	Rank	Block	Slope
**M99-M99**	0.0049	1	**S00–T98**	0.0039
**S00–T98**	0.0027	2	**G00-G99**	0.0036
**G00-G99**	0.0020	3	**H00-H59**	0.0015
**I00-I99**	0.0013	4	**C00-D48**	0.0009
**F00-F99**	0.0007	5	**L00-L99**	0.0006
**K00-K93**	0.0006	6	**O00–O99**	0.0005
**R00–R99**	0.0005	7	**K00-K93**	0.0002
**O00–O99**	0.0005	8	**E00-E90**	0.0001
**L00-L99**	0.0004	9	**D50-D89**	0.0000
**A00-B99**	0.0003	10	**H60-H95**	0.0000
**N00-N99**	0.0003	11	**N00-N99**	0.0000
**P00–P96**	0.0001	12	**P00–P96**	0.0000
**J00-J99**	0.0001	13	**R00–R99**	0.0000
**Q00–Q99**	0.0000	14	**A00-B99**	−0.0002
**D50-D89**	0.0000	15	**Q00–Q99**	−0.0002
**H00-H59**	0.0000	16	**F00-F99**	−0.0011
**C00-D48**	−0.0005	17	**M99-M99**	−0.0014
**E00-E90**	−0.0014	18	**J00-J99**	−0.0014
**H60-H95**	−0.0026	19	**I00-I99**	−0.0040

Additional trends were identified for case management CRs with the highest citation rate since 2015. Musculoskeletal and connective tissue (M00-M99) CRs included an integrative series describing platelet rich plasma injections for sacroiliac joint instability [58], and CRs describing the Chiropractic BioPhysics® methods for posture [59], cervical whiplash [60], and scoliosis [61]. Diseases of the nervous system (G00–G99) CRs described management of postoperative spinal arachnoiditis [62], rehabilitative techniques for a patient with posterior cortical atrophy [63], and integrative management of an incomplete cervical spinal cord injury [64]. Injury, poisoning and certain other consequences of external causes (S00-T98) CRs included multidisciplinary management of inguinal/abdominal muscle strains in a hockey player [65], soft tissue therapy post knee-surgery [66], and diagnostic ultrasonography for lateral ankle injury [67].

Case management CRs in the fourth-fastest growing category, Diseases of the circulatory system (I00-I99), began with the first publication in 1986 [68], with the yearly volume peaking in 2014. Examples include CRs in which chiropractors suspected and referred patients with vertebral artery dissection [69–71], ischemic stroke [72], hemorrhagic stroke [73], upper [74, 75] and lower [76, 77] extremity deep vein thrombosis, abdominal aortic aneurysm [68, 78–81], intracranial aneurysm [82], popliteal aneurysm [83], myocardial infarction [84] and subdural hematoma [85].

The ICD-10 categories with the greatest positive publication trend in the adverse effect topic were Injury, poisoning and certain other consequences of external causes (S00-T98) and Diseases of the nervous system (G00-G99). In contrast, Diseases of the circulatory system (I00-I99) had the greatest negative trend.

### Pediatric cases

Only 138 CRs (12%) included a pediatric subject. There was an “M”-shaped pattern of both the volume and percentage of pediatric CRs with peaks in 1989 and 2010.

### Adverse vs. case management topic

**Fig. 4:**
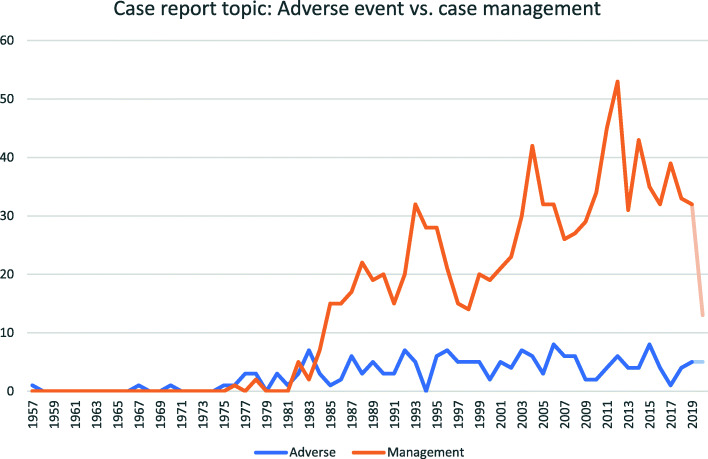
Yearly number of adverse effect or case management case reports. Data from 2020 are de-emphasized as it may not be accurate (see Limitations)

There were 187 CRs describing adverse effects and 989 CRs describing case management (Fig. 4). The number of yearly chiropractic case management CRs irrevocably surpassed those describing adverse effects in 1984, and since 1990 has outnumbered adverse effect CRs in a ratio of 6:1, with a mean 28.5 case management CRs per year and 4.6 adverse effect CRs per year since 1990. The greatest yearly volume of chiropractic case management CRs was 53, in 2012. The 5-year mean percentage of adverse effect CRs has decreased from 77% in 1980 to 17% in 1990 to 15% in 2020.

### Spine vs. non-spinal focus

**Fig. 5:**
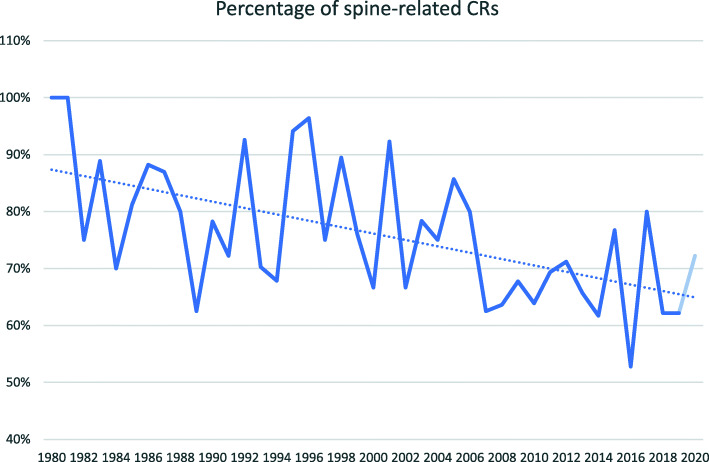
The trend in spine-related case reports, starting at the first year of consecutive publications without zero points (gaps). Data from 2020 are de-emphasized as it may not be accurate (see Limitations)

There was an overall gradual decrease in the percentage of spine-related CRs, and conversely, an increase in non-spine-related CRs (Fig. 5). The 5-year mean proportion of spine-related CRs has reduced from 92% in 1980 to 79% in 1990 to 68% in 2020.

### Citation metrics

Analysis of citation data revealed that 89% of CRs were cited. The median total citations per paper was 7.0, with a mean of 13.0 ± 20.2. The median citations per year was 0.6, with a mean of 0.9 ± 1.5. Six of the ten CRs with the greatest citations per year were published since 2010, with each describing case management (Table 4).

**Table 4:** The ten most frequently cited case reports

Author	Year	Title	Citations per year	Journal
Furmli et al	2018	Therapeutic use of intermittent fasting for people with type 2 diabetes as an alternative to insulin	27.0	BMJ Case Rep
Rubin	2010	Triad of spinal pain, spinal joint dysfunction, and extremity pain in 4 pediatric cases of “Wii-itis”: A twenty-first century pediatric condition	20.5	J Chiropr Med
Moore	2004	Upper crossed syndrome and its relationship to cervicogenic headache	12.8	J Manipulative Physiol Ther
Blum	2002	Chiropractic and pilates therapy for the treatment of adult scoliosis	12.4	J Manipulative Physiol Ther
Marshall et al	2015	The role of the cervical spine in post-concussion syndrome	10.4	Phys Sportsmed
Hammer	2008	The effect of mechanical load on degenerated soft tissue	9.7	J Bodyw Mov Ther
Ko et al	2017	Case series of ultrasound-guided platelet-rich plasma injections for sacroiliac joint dysfunction	8.7	J Back Musculoskelet Rehabil
Ferguson et al	2014	A case of early sports specialization in an adolescent athlete	8.5	J Can Chiropr Assoc
Miners et al	2011	Chronic Achilles tendinopathy: A case study of treatment incorporating active and passive tissue warm-up, Graston Technique®, ART®, eccentric exercise, and cryotherapy	7.8	J Can Chiropr Assoc
Hammer et al	2005	Treatment of a case of subacute lumbar compartment syndrome using the Graston technique	6.1	J Manipulative Physiol Ther

### Journal metrics

Chiropractic CRs were published in 186 unique journals. The majority of chiropractic CRs (852 CRs or 72% of total), regardless of topic or focus, were published in three journals, the *Journal of Manipulative and Physiological Therapeutics*, the *Journal of the Canadian Chiropractic Association*, and the *Journal of Chiropractic Medicine*. Chiropractic journals had published eight adverse effect topic CRs representing 0.9% of their total CRs while medical journals published 164, representing 85% of their total CRs. The medical journal with the greatest number of published chiropractic CRs was *Neurology*, which published six, all of which described adverse effects.

**Fig. 6:**
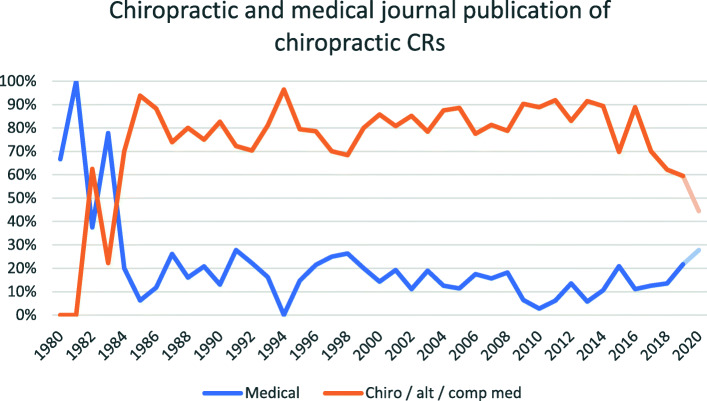
Medical and chiropractic (also including alternative and complementary medicine) journal types shown as a percentage of total publications by year, starting at the first year without zero points / gaps. Data from 2020 are de-emphasized as it may not be accurate (see Limitations)

Prior to the 1980s most chiropractic CRs were published in medical journals, with 79% of these CRs describing an adverse effect. From 1984 to 2020 most chiropractic CRs were published in chiropractic-focus journals (Fig. 6). Publication in chiropractic journals has reduced as a percentage of total CRs since 2017 (70% in 2017, 62% in 2018, 59% in 2019).

**Fig. 7:**
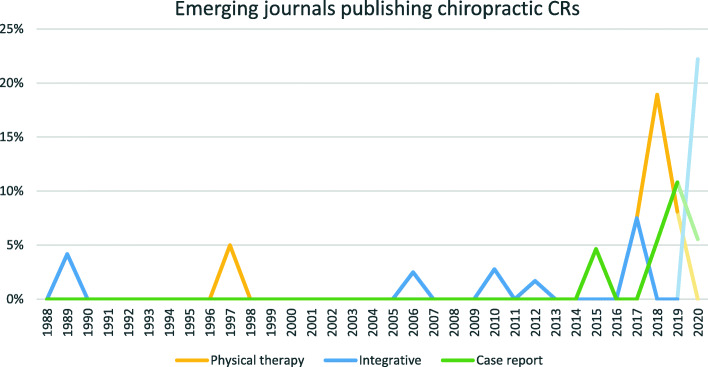
Journal types with the largest positive slope, as a percentage of total CR volume. Data from 2020 are de-emphasized as it may not be accurate (see Limitations)

Since 2015, three journal types have emerged with increasing CR volume: physical therapy, integrative medicine, and dedicated CR journals (Fig. 7). From 2018 to 2020, these have combined to publish up to 28% of chiropractic CRs. The most published physical therapy journal was *J Phys Ther Sci* (13 of 16 in that journal type), integrative medicine was *EXPLORE (NY)* (4 of 11), and CR journal was tied between *AME Case Rep* and *BMJ Case Rep* (3 each of 9).

H-indices were readily available using SCImago for 163 out of 186 Journals. The remainder were calculated via Google Scholar using Harzing’s Publish or Perish.

### Author metrics

There were 2191 unique authors of CRs with a mean 1.5 ± 1.9 and median of 1 CR per author. The most prolific authors were affiliated with chiropractic schools in the USA, Canada, and Australia (Table 5).

**Table 5:** Most prolific authors of chiropractic case reports

Authors	Case reports	H-index	Country	CR Range	CR citations	CR citations per year	Affiliations (in addition to private practice, if present)
Kettner N	48	27	USA	1992–2019	393	39	Logan College of Chiropractic
Cassidy JD	32	46	Canada	1984–1995	157	5	University Hospital, University of Saskatchewan
Taylor JA	18	11	USA	1990–2013	87	11	Los Angeles College of ChiropracticNew York Chiropractic CollegeD’Youville College, Department of Chiropractic
Alcantara J	17	13	USA	1996–2012	218	14	International Chiropractic Pediatric AssociationGonstead Clinical Studies SocietyPalmer College of Chiropractic West
Mierau D	17	8	Canada	1985–1996	41	2	University Hospital, University of Saskatchewan
Harrison DE	17	41	USA	2004–2019	142	33	Chiropractic Biophysics, Non Profit, Inc.
Pollard H	15	36	Australia	1995–2010	337	22	Macquarie University School of Chiropractic
Stern P	14	13	Canada	1994–2014	184	22	Canadian Memorial Chiropractic College,
Chu EC	13	2	China	2017–2020	12	9	New York Chiropractic & Physiotherapy Centre, New York Medical Group
Emary P	13	6	Canada	2009–2017	55	8	None listed
Plaugher G	13	12	USA	1993–2004	208	10	Palmer College of Chiropractic WestLife Chiropractic College West
Oakley PA	13	10	Canada	2017–2019	53	27	Chiropractic Biophysics, Non Profit, Inc.

### Integration metrics

**Fig. 8:**
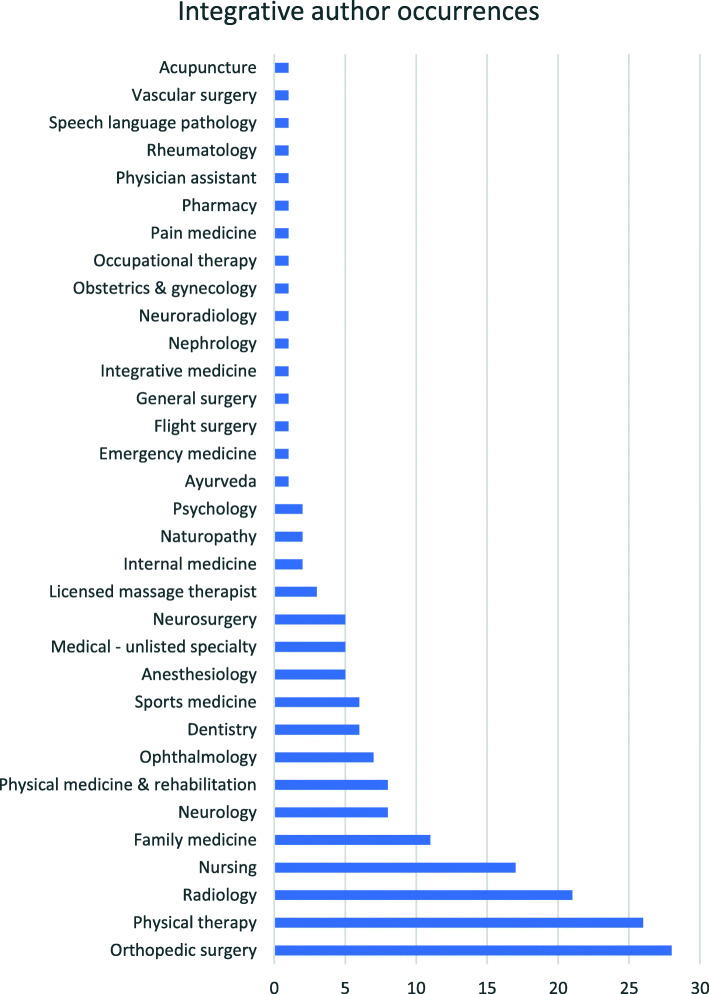
Incidences of non-chiropractic authors in chiropractic CRs. Note that some CRs had more than one type of non-chiropractic author, which was included within the count

There were 158 CRs with integrative authorship teams which represented 13% of all CRs. Most of these described case management type CRs (98% of integrative CRs), while three described adverse effects (2%) [86–88]. The most frequent integrative teams included a chiropractor with another author specializing in orthopedic surgery, radiology, physical therapy, or nursing (Fig. 8). There were several allied health professions and medical specialties not represented including allergy and immunology, anatomic and clinical pathology, cardiology, dermatology, gastroenterology, hematology, infectious disease, oncology, pediatrics, plastic surgery, psychiatry, and urology.

**Fig. 9:**
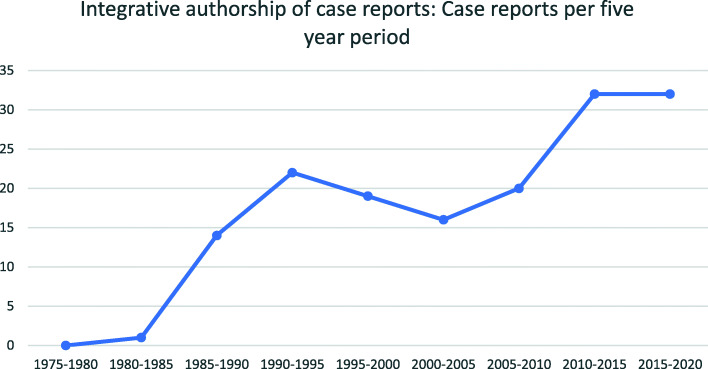
Total number of chiropractic case reports with an integrative authorship team per five-year period

There was an overall increase in CRs with integrative authorship since the first was published in 1984 [89]. There was a temporary downturn between 1995 and 2005 (Fig. 9). The proportion of integrative CRs has not increased since the 1990s. Integrative CRs made up 17% of CRs from 1990 to 1995, and 17% from 2015 to 2020.

### Title metrics

The mean number of words in CR titles was 11.7 ± 4.8, with a median of 11, while 52% of CRs had a colon in the title, and 52% mentioned “case” in the title.

### Logistic regression

Multiple variables were independent predictors of citation counts (Tables 6 and 7). Case reports dealing with Certain conditions originating in the perinatal period (P00-P96) had 13.2 times the odds of receiving more than the median value (0.6) of citations per year, and 5.4 times the odds of having more than the median value (7) of citations. Certain infectious and parasitic diseases (A00-B99) CRs had 1.6 times the odds of having more than the median value of citations. The categories Diseases of the blood and blood-forming organs and certain disorders involving the immune mechanism (D50-D89), Neoplasms (C00-D48), and Symptoms, signs and abnormal clinical and laboratory findings, not elsewhere classified (R00-R99) were independent predictors of fewer citations.

**Table 6:** Logistic regression for citations per year over the median value (> 0.6)

Variable	Odds ratio (95% CI)	***p***-value
> 11 words in title	0.61 (0.47–0.78)	< 0.001
H-index	1.01 (1.00–1.01)	< 0.001
ICD-10 category		
ICD M00–M99	(reference)	
ICD D50-D89	0.56 (0.32–0.99)	0.046
ICD P00-P96	13.22 (2.26–77.34)	0.004
Journal type		
Chiropractic and alternative / complementary medicine	(reference)	
Physical therapy	35.89 (1.96–656.19)	0.016
Integrative	24.26 (1.50–393.54)	0.025
Dental	32.26 (1.71–606.99)	0.020

**Table 7:** Logistic regression for total citations over the median value (> 7)

Variable	Odds ratio (95% CI)	***p***-value
H-index	1.02 (1.01–1.02)	< 0.001
“case” word stem in title	1.55 (1.06–2.27)	0.025
Multidisciplinary authorship (Ref. single-discipline)	0.66 (0.45–0.98)	0.037
Case management topic (Ref. adverse effect)	2.53 (1.11–5.77)	0.027
ICD-10 category		
ICD M00–M99	(reference)	
ICD A00-B99	1.55 (1.03–2.34)	0.035
ICD C00–D48	0.23 (0.07–0.78)	0.018
ICD P00-P96	5.44 (1.20–24.65)	0.028
ICD R00-R99	0.47 (0.23–0.97)	0.041

Physical therapy (OR 35.9), integrative (OR 24.3), and dental journals (OR 32.3) had greater odds of a citation rate greater than the median value. The title word-stem “case” in the title was a predictor of increased citation rate (OR 1.6) while a lengthy title (> 11 words) was a predictor of reduced total citations. Case management topic CRs had greater odds (OR 2.5) of having more citations than the median value, while multidisciplinary authorship was a negative predictor (OR 0.7).

H-index had a negligible odds ratio for affecting citations, while spine focus, having a pediatric subject, and a colon punctuation in the title were not statistically significant predictors of citations.

## Discussion

### Emerging topics

#### Neuromusculoskeletal CRs

The three categories of chiropractic CRs with the greatest publication volume were also the fastest growing among those with a case-management topic: Diseases of the musculoskeletal system and connective tissue (M00–M99), Injury, poisoning and certain other consequences of external causes (S00–T98), and Diseases of the nervous system (G00–G99). According to a 2014 survey, the most common conditions managed by American chiropractors include disorders of the joints, nervous system, and muscles [90], conditions which fit into these ICD-10 categories.

Further analysis of Diseases of the musculoskeletal system and connective tissue (M00–M99) showed that more recent CRs had a sports-medicine or rehabilitative non-spine-related focus. Case reports in Diseases of the nervous system (G00–G99) highlighted unique approaches to management or uncommon presentations of neurological illnesses.

#### Vascular diagnosis CRs

There was an increase in CRs describing potentially life-saving identification of vascular pathology (ICD I00-I99) preceding manual therapy interventions, such as vertebral arterial dissection in-progress [69–71]. Authors of vascular diagnosis CRs pointed out the difficulty in diagnosing vertebrobasilar arterial pathology [69] and inadequacy of provocative physical maneuvers [91]. None of these CRs described the use of the newer Head Impulse, Nystagmus, and Test of Skew (HINTS) examination [92]. In contrast to the emergence of these vascular diagnosis-oriented CRs, adverse effect I00-I99 CRs had the strongest negative publication trend of any ICD-10 category.

A recent systematic review concluded there was no convincing evidence of a link between chiropractic spinal manipulation and cervical arterial dissection, which had been hypothesized in prior studies and adverse effect CRs [93]. Vascular diagnosis CRs provide contextual evidence to support the hypothesis [94] that an epidemiologic association between chiropractic visits and the onset of vascular pathology could be explained by patients presenting to the chiropractic office with pre-existing occult vascular-related pathology. Ultimately, case-control study designs are better suited to study rare adverse outcomes.

#### Non-spinal musculoskeletal topics

Some chiropractic research agendas have prioritized the research of the efficacy of chiropractic care for musculoskeletal conditions outside of the spine, including sports-related soft-tissue and extremity disorders [95, 96]. Of the ten case management CRs in M00-M99 with the highest citation rate, seven described instrument-assisted soft tissue manipulation for treating extremity injuries [97–103], six of which involved athletes. Similarly, in S00-T98, eight of the ten case management CRs with the highest citation rate described sport-related injuries, and only half of these had a spinal focus. Sports medicine appears to be an emerging area of chiropractic CRs.

#### Concussion-related disorders

There were seven CRs describing post-concussion syndrome or traumatic brain injury, all of which were published since 2014 [104–110]. Because there is no unifying ICD-10 group for these diagnoses, these CRs were split into Diseases of the nervous system (G00–G99, 1 CR), Injury, poisoning and certain other consequences of external causes (S00–T98, 2 CRs), and Mental and behavioural disorders (F00-F99, 4 CRs). A test of recoding these under F00-F99 did not change the position of F00-F99 from the 5th fastest growing ICD-10 category.

### Gaps in the literature

#### Commonly seen conditions

Comparison of CR data to a survey from 2014 [90] revealed a discrepancy between the ICD-10 publication volumes and frequency of diagnoses seen by American chiropractors (Table 8). Certain conditions seen on a monthly basis had a low publication volume while in contrast, congenital anomalies (Q00-Q99) had a high volume relative to being seen only on a yearly basis [90].

**Table 8:** ICD-10 categories with a discrepancy between CR volume and frequency seen in a chiropractic setting

ICD-10 Block	CR volume	Example condition(s)	Average frequency seen [90]
H60–H95	7	• Vertigo / loss of equilibrium	Monthly
E00-E90	13	• Diabetes / metabolic syndrome • Nutritional disorder	Monthly
O00-O99	12	• Pregnancy related conditions	Monthly
Q00-Q99	54	• Hereditary multiple exostosis	Yearly

#### Pregnant women, pediatrics, and infants

Case management CRs in specific populations including pregnant women, and pediatric patients, including infants, had a low publication volume, represented by ICD-10 categories O00-O99 and P00-P96. One possible explanation is that authors may publish these in non-PubMed indexed journals such as the *Journal of Clinical Chiropractic Pediatrics*.

#### Non-musculoskeletal topics

Non-musculoskeletal ICD-10 topics had a low publication volume. Among these, specific CRs were distinct as having little additional evidence supporting the use of chiropractic spinal manipulation above the CR level. These included gastroesophageal reflux [53, 111], constipation [112, 113] and pruritis [114]. In contrast, CRs documenting improvements in asthma post-spinal manipulation may be limited because this topic has been explored by larger study designs including randomized controlled trials [115].

### Collaboration in chiropractic

Multiple international research agendas have prioritized collaborative research activities [95, 116–119]. The increasing publication volume of integrative CRs is an example that this is occurring.

Although integrative authorship was a negative predictor of CR citations in our regression model, there may be less-tangible benefits to multidisciplinary collaboration. Citation tracking of integrative author teams demonstrated that certain teams had cooperated later to co-publish larger studies (Table 9), which were often on different topics. This suggests that an integrative authorship CR may be a gateway to conducting research with a higher level of evidence, irrespective of topic.

**Table 9:** Examples of collaborative research case reports and larger subsequent studies

First case report reference	Larger study (only one example shown if multiple)
**Plaugher** G, (DC) **Bachman** TR (MD, rheumatology). Chiropractic management of a hypertensive patient. Journal of manipulative and physiological therapeutics. 1993 Oct 1;16 (8):544–9.	Menke JM, **Plaugher** G, Carrari CA, Coleman RR, Vannetiello L, **Bachman** TR. Likelihood-Evidential Support and Bayesian Analysis on a Prospective Cohort of Children and Adolescents with Mild Scoliosis Under Chiropractic Management. Journal of the Arizona-Nevada Academy of Science. 2007 Jan 1:99–111.
**Dreyfuss** P (MD, physiatry), **Michaelsen** M (DC), Horne M. MUJA: manipulation under joint anesthesia/analgesia: a treatment approach for recalcitrant low back pain of synovial joint origin. Journal of manipulative and physiological therapeutics. 1995 Oct;18 (8):537–46.	**Dreyfuss** P, **Michaelsen** M, Pauza K, McLarty J, Bogduk N. The value of medical history and physical examination in diagnosing sacroiliac joint pain. Spine. 1996 Nov 15;21 (22):2594–602.
**Skaggs** CD (DC), Winchester BA, Vianin M, **Prather** H (DO, sports medicine). A manual therapy and exercise approach to meralgia paresthetica in pregnancy: a case report. Journal of Chiropractic Medicine. 2006 Sep 1;5 (3):92–6.	**Skaggs** CD, **Prather** H, Gross G, George JW, Thompson PA, Nelson DM. Back and pelvic pain in an underserved United States pregnant population: a preliminary descriptive survey. Journal of manipulative and physiological therapeutics. 2007 Feb 1;30 (2):130–4.
**Aspegren** D (DC), Hyde T, **Miller** M. (MD, family medicine) Conservative treatment of a female collegiate volleyball player with costochondritis. Journal of manipulative and physiological therapeutics. 2007 May 1;30 (4):321–5.	**Aspegren** D, Enebo BA, **Miller M**, White L, Akuthota V, Hyde TE, Cox JM. Functional scores and subjective responses of injured workers with back or neck pain treated with chiropractic care in an integrative program: a retrospective analysis of 100 cases. Journal of Manipulative and Physiological therapeutics. 2009 Nov 1;32 (9):765–71.
**Morningstar** MW (DC), **Strauchman** MN (DO, family medicine). Management of a 59-year-old female patient with adult degenerative scoliosis using manipulation under anesthesia. Journal of chiropractic medicine. 2010 Jun 1;9 (2):77–83.	**Morningstar** MW, **Strauchman** MN, Weeks DA. Spinal manipulation and anterior headweighting for the correction of forward head posture and cervical hypolordosis: a pilot study. Journal of chiropractic medicine. 2003 Mar 1;2 (2):51–4.
Seidman MB, **Vining** RD (DC), **Salsbury** SA (RN, PhD). Collaborative care for a patient with complex low back pain and long-term tobacco use: a case report. The Journal of the Canadian Chiropractic Association. 2015 Sep;59 (3):216.	Gudavalli MR, **Salsbury** SA, **Vining** RD, Long CR, Corber L, Patwardhan AG, Goertz CM. Development of an attention-touch control for manual cervical distraction: a pilot randomized clinical trial for patients with neck pain. Trials. 2015 Dec 1;16 (1):259.

### Professional development

Further analysis of the CR database identified multiple chiropractic college presidents who published CRs prior to their presidency, such as Drs. Michael Mestan [120] (New York Chiropractic College), William Morgan [109] (Parker University), William Meeker [121] (Palmer College of Chiropractic), John Scaringe [122] (Southern California University of Health Sciences), as well as top researchers in the field (e.g. Simon French) [123], and developers of postgraduate training (e.g. Donald Murphy) [124].

### Future use of CRs by chiropractors

Case reports are more accessible, achievable, and do not require advanced research skills compared to more sophisticated research designs [125]. In this sense CRs may be more attractive to chiropractic practitioners without an additional research degree or training [21, 28]. Writing a CR has become straightforward due to the CARE guidelines, which act as a checklist to ensure relevant information is included in a CR [34]. Adherence to CARE guidelines may also increase the impact of chiropractic CRs, as having “case” in the title independently predicted a greater number of citations in our regression model.

Case reports can serve as a foundation for larger studies such as cohort studies and trials which ultimately improve patient care [125]. Synthesis or review of similar CRs and case series is also possible, which can add to the evidence base from which to make clinical decision-making when there is no higher level of evidence available [1]. Examples of prior syntheses incorporating chiropractic CRs include those focusing on non-musculoskeletal conditions [126], upper extremity conditions [127], and colic [128]. Future syntheses might describe the clinical features of patients presenting to a chiropractic office with a stroke in progress, or the treatment of coexisting cervical spine disorders in patients with post-concussion syndrome.

It was unclear why certain chiropractors were so prolific in publishing CRs. Future research could involve a survey of CR authors to determine their motivations for publishing CRs, interest in publishing certain CR topics or diseases, preference of target journal type, opinion towards integrative authorship, and interest in conducting research with larger study designs.

### Limitations

Aside from the title including “case,” we did not calculate a quality score for the CARE guidelines rubric, which could have enabled us to investigate temporal changes in the quality of reporting CRs. Older case reports may not have “case report” in the title due to editorial style guidelines. As the CARE guidelines recommending this were only recently introduced, title metrics should be interpreted with caution.

Additional case reports may have been identified by searching more databases such as MANTIS/Chirolars and CINAHL. Due to delays or embargos in indexing CRs in PubMed, some of the recent CRs from 2020 were not indexed and therefore not included in the study. Although omission of non-PubMed indexed CRs was done purposefully, it is possible that additional insights could have been gained by including these CRs. While CRs are a large part of chiropractic research, trends in CRs may not be representative of all research types in the chiropractic field. The results of this review could have been affected by heterogeneity of study type, as we included both traditional retrospective CRs, which constituted the majority of included CRs, and prospective or N-of-1 case studies.

It is possible that confounding variables were missed that would have changed the qualitative and quantitative analyses. Dichotomizing cases as “rare” or “not rare” by a prevalence cutoff would have given insight into publication trends and associations for rare conditions. Diagnosis of a rare condition is a justification for publishing a CR and emphasizes the diagnostic process rather than treatment.

This review could have been strengthened by categorizing chiropractic authors by presence of a diplomate or master’s degree in a clinical specialty such as radiology, pediatrics, nutrition, orthopedics/neuromusculoskeletal medicine, or sports. Specialization could be associated with greater publication volume, citation rate, and/or growth of certain CR topics. While only 37% of chiropractors have (or are working towards) a post-graduate diplomate or master’s degree [2], such programs emphasize [129] or require [130] the publication of research, or offer it as an option to attain program credits [131]. In contrast, other research has shown that chiropractors generally lack the training required to conduct clinical research [14].

Data extraction was done independently (RT) and verified by a second author (JD) rather than in duplicate, which could have led to an increased error rate.

## Conclusions

Chiropractic CRs have historically often described spine-related musculoskeletal disorders in journals with a chiropractic focus. Although these are still the predominant themes, this review identified markers of increasing variety of chiropractic CRs, with trends towards a non-spine-related focus and non-chiropractic journals. Vascular-related case management CRs have emerged while those describing adverse effects have declined. Many disease categories and patient populations represent areas of need for chiropractic CRs such as pregnancy and pediatrics. Authors need to be aware of simple editorial features associated with a greater citation impact such as a title structure that follows recommended guidelines. We encourage chiropractors and chiropractic students to publish CRs to fill research gaps. In so doing, we contend that clinical advances will be better understood and well-known, and that the CRs would serve as the foundation for future research studies including randomized controlled trials. Taken together, this effort will increase the relevance of chiropractic medicine to clinicians and improve the health of patients.

## Supplementary Information

**Additional file 1.**

## Data Availability

The datasets supporting the conclusions of this article are available in the Figshare repository, 10.6084/m9.figshare.13591151.

## Abbreviations

CARE: CAse REports
CR: Case report
ICD: International Classification of Disease
ICL: Index to Chiropractic Literature
IF: Impact factor
OR: Odds ratio
SJR: SCImago Journal Rank indicator
